# Laser recrystallization and inscription of compositional microstructures in crystalline SiGe-core fibres

**DOI:** 10.1038/ncomms13265

**Published:** 2016-10-24

**Authors:** David A. Coucheron, Michael Fokine, Nilesh Patil, Dag Werner Breiby, Ole Tore Buset, Noel Healy, Anna C. Peacock, Thomas Hawkins, Max Jones, John Ballato, Ursula J. Gibson

**Affiliations:** 1 Department of Physics, Høgskoleringen 5, NTNU, Norwegian University of Science and Technology, Trondheim 7491, Norway; 2 Department of Applied Physics, KTH Royal Institute of Technology, Roslagstullsbackan 21, Stockholm 100-44, Sweden; 3 Department of Micro- and Nano System Technology, University College of Southeast Norway, Campus Vestfold, Raveien 215 N-3184 Borre, Norway; 4 Optoelectronics Research Centre, University of Southampton, Highfield, Southampton, Hampshire SO17 1BJ, UK; 5 Physics Department, Emerging Technology and Materials Group, Newcastle University, Merz Court, Newcastle NE1 7RU, UK; 6 Department of Materials Science and Engineering and the Center for Optical Materials Science and Engineering Technologies (COMSET), Clemson University, Clemson, SC 29634, USA

## Abstract

Glass fibres with silicon cores have emerged as a versatile platform for all-optical processing, sensing and microscale optoelectronic devices. Using SiGe in the core extends the accessible wavelength range and potential optical functionality because the bandgap and optical properties can be tuned by changing the composition. However, silicon and germanium segregate unevenly during non-equilibrium solidification, presenting new fabrication challenges, and requiring detailed studies of the alloy crystallization dynamics in the fibre geometry. We report the fabrication of SiGe-core optical fibres, and the use of CO_2_ laser irradiation to heat the glass cladding and recrystallize the core, improving optical transmission. We observe the ramifications of the classic models of solidification at the microscale, and demonstrate suppression of constitutional undercooling at high solidification velocities. Tailoring the recrystallization conditions allows formation of long single crystals with uniform composition, as well as fabrication of compositional microstructures, such as gratings, within the fibre core.

## Abstract (web summary)

Using SiGe in the core of optical fibres extends the wavelength range and potential optical functionality, but fabrication challenges exist. Here, Coucheron *et al*. report the fabrication and tailoring of SiGe-core optical fibres using CO_2_ laser irradiation to heat the glass cladding and recrystallize the core.

Optical fibre research has expanded tremendously in recent decades, due to the incorporation of non-traditional materials, such as chalcogenides^1^,^2^ and fluorides^3^, and novel designs, such as photonic crystal fibres^4^,^5^,^6^. These advances have extended the accessible wavelength range and increased the number of potential applications, particularly in the infrared^7^ region. Increases in functionality are also enabled by the combination of optical and electrical phenomena in a single fibre, approached either from the use of hybrid structures^8^, or core materials such as semiconductors^9^,^10^, which respond to both inputs. Semiconductors also have very large non-linear optical coefficients^11^, important for all-optical processing, and are transparent through large parts of the infrared and terahertz spectral regions.

Fibres with elemental semiconductor core materials were first reported in 2006 (ref. 12), initially fabricated by using a high-pressure chemical vapour deposition method to fill the internal channels of glass capillaries. Soon afterwards a more conventional fibre-tower drawing method was demonstrated^13^, which has since been widely adopted by the community^14^,^15^,^16^. Both methods have been adapted to incorporate a range of materials within the core, and silicon, germanium and compound semiconductor fibres have been fabricated^17^,^18^,^19^,^20^ for use in applications from sensing to electro-optic modulation and photovoltaic conversion^9^,^21^,^22^,^23^,^24^,^25^. Significantly, the silica glass cladding can also serve as a microcrucible for modifying the core material, either during or after fabrication. As examples, reactive formation of silicon fibres from aluminium cores^15^ and production of minute silicon spheres by heat treatment^26^ has been reported.

Typically, the cores of as-drawn fibres are polycrystalline, with grain sizes that decrease in size with decreasing core diameter^27^. Transverse grain boundaries, which accumulate deleterious impurities and reduce light transmission, represent a particular challenge for the micrometre-core fibres typically required for optical applications. In addition, in an alloy system, non-uniform composition could lead to excess scattering due to the refractive index variations. Rapid thermal annealing and related techniques^28^ have been used to crystallize insulator-encapsulated alloy thin^29^,^30^ and thick^31^ films, and similar processing has been shown to improve overall crystallinity of semiconductor core fibres^32^. Laser processing, for which the fibre geometry is particularly well suited, as established in the case of conventional glass fibres^33^,^34^,^35^, combines a small heat zone and large thermal gradients with fine motion and temperature control. The cylindrical geometry, with a large surface to volume ratio, allows efficient convective cooling and optical access to a small heating volume, essential for establishing controlled, extreme thermal gradients. Direct, selective heating of the core using a tightly focused visible laser beam (to which the glass cladding is transparent) has been shown to both crystallize and alter the local stress and related properties of an elemental silicon fibre core after fabrication^36^. Infrared laser treatment, in which the glass absorbs the energy, has recently been used to recrystallize silicon fibres and reduce their optical loss^37^.

SiGe alloy cores offer an extended range of optoelectronic functionalities, since their bandgap and index of refraction can be tailored by changing the composition. The alloy can form a single-phase solid solution, crystallizing over the temperature range 938–1,414 °C, depending on the Si:Ge ratio. However, the equilibrium phase diagram, (Fig. 1a; ref. 38) has a large separation between the solidus and liquidus, and when the material solidifies at finite rates, the initial precipitate is silicon-rich relative to the melt. Diffusion is too slow in the solid state to allow equilibration, and the composition of the solid acquires spatial gradients, as illustrated in Fig. 1b for the case of multiple nucleation sites. These stochastic variations are beneficial for some applications, and can also reveal the thermal history of the solidification process^39^,^40^, as the last regions that solidify will be Ge-rich. Ordered variations in the composition of SiGe, particularly in thin film form, have been used for strain engineering^41^, quantum cascade lasers^42^ and infrared filters^43^.

For optical transmission, compositional homogeneity is preferred. More uniform samples, as illustrated in Fig. 1c can be made by the traveling liquid-zone method^44^,^45^, where a dynamic equilibrium is established between the silicon provided by the supply end of the rod and that deposited in the growing crystal. However, even in directional recrystallization, constitutional undercooling can lead to formation of dendrites and locally non-uniform composition if the temperature gradients are small or the solidification velocity is above a critical value^46^.

Here we report on the fabrication of silica-clad fibres with a SiGe-core. CO_2_ laser treatment, during which the silica cladding is heated using strongly absorbed infrared radiation and the core is melted by thermal conduction, is used to recrystallize the fibre cores, while softening the glass to avoid inducing large strains. The softening of the glass also allows shaping or tapering of the fibre during recrystallization. As-drawn fibres of SiGe manifest the effects described by the classic Tiller model^46^ of undercooling. We demonstrate that, using laser treatment, both compositionally uniform and structured semiconductor alloy optical fibre cores can be made. We discuss the recrystallization processes in the light of classical solidification theories and the steep thermal profile present during the laser heat treatment.

## Results

### As-drawn fibres

**Figure 2:**
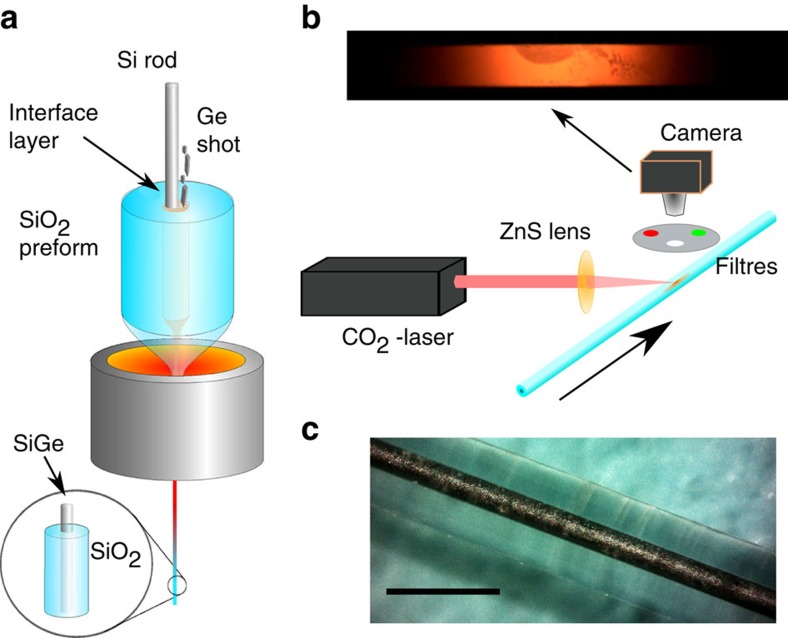
Fabrication and processing of fibres. (**a**) A schematic of the fibre drawing process used in this work, and (**b**) schematic of the laser recrystallization set-up. The inset shows an image from the camera. (**c**) A photomicrograph of a SiGe-core fibre (Scale bar, 1 mm).

We used the fibre draw method illustrated in Fig. 2a to fabricate glass-clad SiGe-core fibres with a core diameter of 130 μm and a composition of 6 at% Ge. Some fibres were subsequently annealed using the procedure illustrated in Fig. 2b. A typical large-core fibre is shown in Fig. 2c. Additional fibres with germanium concentrations of 25–40 at% were made using the procedure described by Nordstrand^47^. Smaller-core fibres of all compositions were made by redrawing larger diameter fibres. After fibre fabrication, the crystal structure and germanium distribution were examined to infer the non-equilibrium nature of the crystallization process during drawing.

In the as-drawn fibres, there was little variation in the average composition over macroscopic lengths. Despite the constant average composition in the fibres, significant local variations in composition were observed in the as-drawn material consistent with compositional undercooling and the breakdown of a planar solid–liquid growth interface (as described by Tiller and others^48^,^49^). These variations indicated that the drawing process took place above the critical growth velocity, but below the velocity for partitionless solidification^50^.

**Figure 3:**
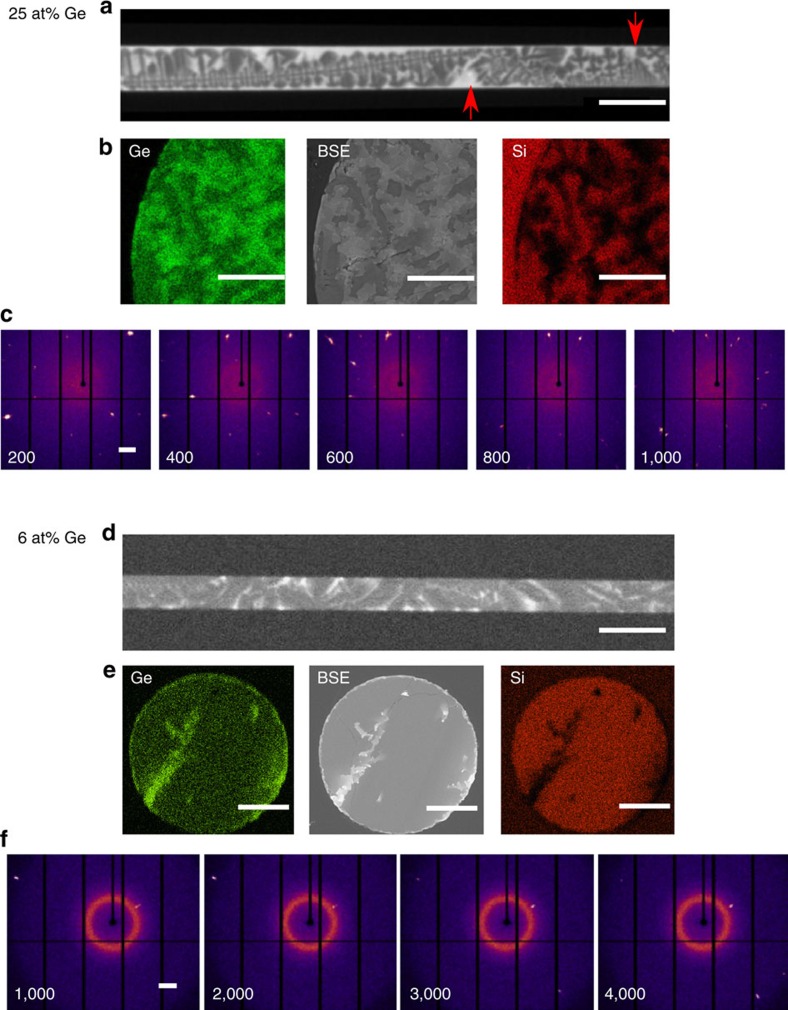
Composition and structure of SiGe-core fibres. (**a**–**c**) 25 at% Ge fibre (**a**) XCT image of dendritic structures, showing a large grain, and red arrows indicating grain boundaries. (**b**) Cross-sectional BSE image (grey), EDX compositional maps for Ge (green) and Si(red), and (**c**) X-ray diffraction patterns of a polycrystalline region as a function of axial position (numbers shown are relative position in μm). Note that there are numerous peaks, the placement and number of which varies from frame to frame. (**d**–**f**) 6 at% Ge fibre (**d**) XCT image showing Ge segregation without dendrite formation (**e**) Cross sectional BSE image (grey), EDX compositional maps for Ge (green) and Si(red); and (**f**) truncated X-ray diffraction patterns of a fibre as a function of axial position (numbers are relative position in micrometre). Fewer peaks are seen, and the positions are constant over larger distances. The fibre has a grain boundary between 2,000 and 3,000 μm, as revealed by the sudden change in the pattern. Scale bar, 200 μm (**a**,**d**), 20 μm (**b**), 40 μm (**e**) and 1 Å^−1^ (**c**,**f**). X-ray diffraction patterns in **c**,**f** are rotated 90° counter-clockwise to match the shown fibre orientation.

Figure 3 shows experimental results from the fabrication of 25 at% (Fig. 3a–c) and 6 at% (Fig. 3d–f) Ge fibres. We used large-core fibres for the materials characterization so that scanning electron microscopy (SEM), X-ray diffraction and tomography could all be performed on the same fibre samples. Figure 3a,d shows the compositional variations for as-drawn fibres of two average compositions, imaged non-destructively using X-ray computed tomography (XCT), and Fig. 3b,e shows cut and polished cross-sections imaged using energy dispersive X-ray spectroscopy (EDX) and backscattered electron (BSE) techniques. In both the (contrast-inverted) XCT and BSE images, Ge-rich areas appear brighter, due to the higher absorption and higher atomic number, *Z*, respectively. The EDX images (Fig. 3b,e; coloured images) provide identification of the components, and quantitative analysis indicates that the oxygen content is less than ∼3 at%. As fibres were drawn in an air ambient, the oxygen likely originates from the atmosphere or possibly from reaction of germanium with the CaO interface modifier. The latter would release atomic oxygen that would then be free to diffuse through the core.

With 25 at% Ge, the constitutional undercooling is severe, and dendritic silicon-rich structures are developed, as seen in Fig. 3a and Supplementary Movie 1. Grain boundaries (indicated by red arrows in Fig. 3a) are clearly delineated by abrupt orientation changes in the dendrites and regions of high-germanium content that solidify at the termination of each grain. The latter are similar to the Ge-rich terminal caps in nanowires^51^ and microencapsulated SiGe films^30^. The orientation of an unusually long dendrite can be seen to vary several degrees over a length of ∼1 mm. Dendritic structures are not desired for most optical applications, but may be favourable for thermoelectric^52^ or photovoltaic^53^ uses, where enhanced phonon and light scattering could be an advantage. X-ray diffraction of these Ge-rich fibres, performed with a 0.2 mm probe, revealed complex patterns consistent with multiple crystal orientations within the scattering volume at most axial positions (Fig. 3c).

In the 6 at% Ge fibres, initial precipitation of the silicon-rich material occurs along the outer regions, and Ge-rich features develop in the last-to-solidify regions in a classic example of coring, as illustrated in Fig. 3d,e and shown in Supplementary Movie 2. The distribution of germanium and the axial features in Fig. 3d,e suggest that the solidification proceeds smoothly, primarily from the cladding interface towards the centre, with little undercooling until the remaining liquid has a high enough germanium content to destabilize the growth front. Despite the large compositional variations (3–35 at% Ge) observed within the 6 at% (average composition) Ge fibres, crystalline grains extend through the entire fibre diameter, as demonstrated by X-ray diffraction (Supplementary Fig. 1). Electron backscattered diffraction (EBSD) of cross-sections confirmed the absence of transverse grain boundaries (Supplementary Fig. 2).

All as-drawn fibres were polycrystalline, with grain sizes 0.2–1 mm in length as shown by X-ray diffraction patterns (Fig. 3c,f), acquired at different positions for a fixed projection angle. X-ray diffraction data for rotation of fibres at a fixed axial position (Supplementary Fig. 1) revealed multiple crystals within the cross-section of the high-Ge fibres and a single grain for the low germanium fibres in most of the 0.2 mm analysis windows.

### Recrystallization and compositional homogenization

For optical and electrical applications, controlled (and usually, uniform) composition is desired. One approach to achieve this is to use laser-induced recrystallization to alter the core structure. While the energy in visible-wavelength laser processing was deposited directly into the core after traversing the transparent glass cladding^36^, in this work the silica glass was heated and softened using radio frequency modulated quasi-continuous wave infrared radiation. The absorption depth in the silica is ∼20 μm at the incident wavelength^54^, so melting of the core is via thermal conduction from the surface-heated glass. The emissivity of the core far exceeds that of the glass in the visible-wavelength range, allowing real-time observation of the melting and resolidification process through the transparent cladding. The emission collected by the camera originates in the near-surface region of the fibre core, due to the large absorption coefficient of the semiconductor at visible wavelengths.

**Figure 4:**
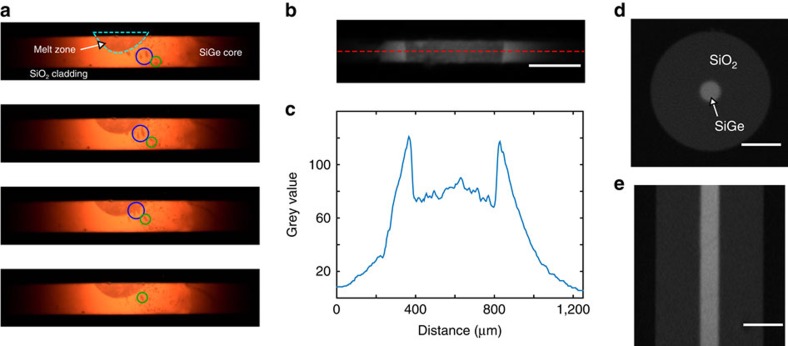
Laser recrystallization of 6 at% Ge fibres. (**a**) Frames from CCD video showing Ge-rich liquid flowing from the untreated region to the laser-induced melt zone. Green and blue circles highlight droplet motion. (Frames are stretched 135% in the vertical direction for clarity.) (**b**) Image of melt zone penetrating the entire core. White spots are emission from interface layer inhomogeneities. (**c**) Intensity profile from the emission at the location shown by the red dashed line in **b**, showing a temperature increase towards the centre, and uneven emission from the interface layer. The large drop in emissivity between the solid and the liquid allows observation of the interface. (**d**) XCT cross-section after recrystallization (background is removed from around the fibre) and (**e**) XCT side view after recrystallization. Scale bar, 200 μm.

The initial inhomogeneity of the fibre, with inclusions of high-germanium content, leads to an interesting phenomenon on initial melting of the fibre core, as shown in Fig. 4a and in Supplementary Movie 3. As the laser power is increased, the germanium-rich regions melt first. Silicon is more soluble in this liquid at the higher temperatures close to the melt zone, and this provides a mechanism for germanium-rich liquid to move toward the beam centre, dissolving silicon at the hot side of the droplet, and precipitating it at the lower temperature side. The movie shows germanium-rich liquid streaming towards the high temperature region and being incorporated into the melt. The melt zone gathers a higher concentration of germanium than the average composition in the fibre through this mechanism.

Directional recrystallization of fibres was undertaken by establishing a melt zone across the entire core, as shown in Fig. 4b. Emission intensity profiles were recorded as shown in Fig. 4c, where a large drop in emissivity of the liquid compared to the solid, as reported for pure silicon^55^, can be seen, and from which the extent of the melt zone can be determined. Changing the focus and power of the laser allowed control of the width of the melt zone from ∼100 to 1,000 μm. Images taken through two narrowband filtres allowed the temperature gradient to be estimated in the vicinity of the solidification front (Supplementary Note 1 and Supplementary Figs 3 and 4), yielding 1–2 × 10^4^ K cm^−1^. This is a factor of 10^3^ higher than gradients typical for bulk crystallization, but much lower than those in pulsed laser film recrystallization experiments^56^.

### Critical velocity

The small molten volume makes the fibres an ideal testbed for measurement of the critical growth velocity^46^ in reduced dimensions, as well as for investigations of the stabilizing effect of capillarity proposed by Mullins and Sekerka^48^. The latter model includes prediction of a higher critical velocity for small core diameters, in addition to the thermal gradient and concentration effects included in the Tiller criterion^46^:


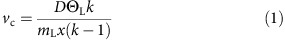


where *v*_c_ is the critical velocity, *D* is the diffusion coefficient in the liquid, *k* is the segregation coefficient, 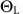 is the temperature gradient in the liquid, *m*_L_ is the slope of the liquidus and *x* is the germanium composition in the liquid.

The growth (solidification) velocity was controlled by the translation rate of the CO_2_ laser (1–1,000 μm s^−1^), and two different compositions (6 at%, 40 at% Ge) were investigated, with different core diameter (130–250 μm, and 15 μm) fibres for both compositions. The 6 at% Ge fibres had critical velocities of 200 μm s^−1^ for large-core diameters, which compares well with the 165 μm s^−1^ value calculated (Supplementary Note 2 and Supplementary Figs 5 and 6) on the basis of our temperature gradient estimates. In accordance with the Tiller model, higher germanium concentrations resulted in lower critical velocities (10 μm s^−1^ for the 250 μm cores), and the fluctuating deviations from a planar growth front could be seen (Supplementary Fig. 7). Fibres of both concentrations with 15 μm cores had critical velocities that were an order of magnitude higher, suggesting the stabilizing role of small core dimensions^48^. However, the diameter for absolute stability was not reached, as evidenced by the inhomogeneity of as-drawn small-core fibres. Details of the critical velocity experiments and data analysis can be found in Supplementary Note 2.

### Single-crystal formation

The fibre shown in Fig. 4d,e (130 μm core 6 at% Ge) was recrystallized at 100 μm s^−1^, below the critical velocity, and was studied in detail using X-ray diffraction. The analysis showed a single crystal with almost constant orientation over the entire analysed length of 7 mm (Supplementary Note 3 and Supplementary Figs 8–11). The rapid translation of the melt zone and the large temperature gradients in the core clearly suppress nucleation and crystallization at locations other than the advancing solidification front. In contrast, a fibre annealed at 10 μm s^−1^ had many qualitatively different diffraction patterns over a similar length, as would be expected for multiple grains. The velocity dependence of grain size is not anticipated by any of the models, as they do not consider nucleation. However, a general picture of the effect of the translation velocity can be formed by considering the competition between formation and growth of nuclei on defects at the core-cladding interface and extension of the growth of the existing crystal.

In detailed experiments on both seeded and unseeded crystallization of water, Heneghan *et al*.^57^ showed that the fraction of samples still in the liquid state after time *t* goes as *e*^*−kt*^, where *k* is a parameterized function of the degree of undercooling and material properties. They further demonstrated that this temporal behaviour holds true for a wide variety of systems, including metallic alloys. If we consider sequential portions of the fibre to be equivalent to a series of samples, the likelihood of a nucleus forming before the passage of the existing crystal phase front depends on the time available, and hence on the velocity of the heat zone through the fibre. At high velocities, the chances of a stable nucleus forming are reduced. In addition, any nuclei that are formed need time to grow inward from the core-cladding interface if they are to compete with the existing crystal in the core. A high velocity also minimizes the time for growth, and favours the formation of large single-crystal regions. In addition, any interfacial roughening or compound formation driven by the laser treatment would result in a higher density of heterogeneous nucleation sites at lower scan velocities, and thus a tendency toward smaller grains.

The recrystallization process did not alter the oxygen content of the cores as determined by EDX, within experimental error. Although the absorption of atomic oxygen in silicon occurs primarily at wavelengths >6 μm, microscopic oxygen-related clusters of impurities may lead to scattering and thus a degradation of the optical properties of the material, even when a SiGe crystalline core is formed.

High-quality crystals are a necessary (though not sufficient) condition for good optical properties and preliminary measurements of the transmission losses of several large-core recrystallized fibres with 6 at% Ge were therefore made. Before the laser treatment, losses were in excess of 20 dB cm^−1^, as would be expected from the XCT results, which imply large refractive index inhomogeneity. In the absence of any optimization of the interface layer, the fibre cores are continuous, but annealed large-core fibres show some oxygen inclusion, and large scattering features at the interface due to chemical interactions (Supplementary Figs 4 and 5). Even in the presence of these imperfections, the losses (12 dB cm^−1^ at 1,550 nm and 9.7 dB cm^−1^ at 2,000 nm) measured for a fibre with a 130 μm core, suggest that with attention to optimizing the bulk material and interface defects, these fibres have a promising future.

### Microarchitecture control

**Figure 5:**
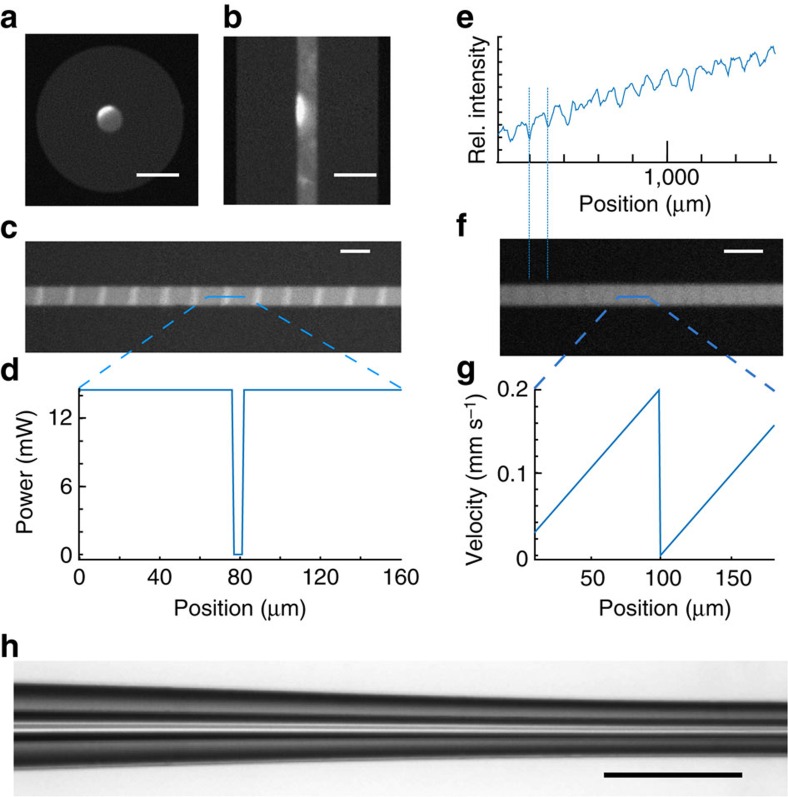
Microstructuring of 6 at% Ge fibres during recrystallization. (**a**) XCT cross section of a Ge-rich lobe formed by melting the core and gradually reducing the laser power density, (**b**) XCT side view of the centre of the Ge-rich region, (**c**) Ge-rich grating formed in the fibre core by periodically interrupting the laser beam. The angle of the grating is due to the asymmetric heating and resultant tilted solidification boundary. (**d**) Power versus distance for the germanium grating process. (**e**) Intensity profile for the grating in **f**, where the dashed lines highlight the alignment. The upward slope is an unsubtracted background effect. (**f**) Si-rich regions formed by periodic variation of the velocity during recrystallization and (**g**) velocity profile during formation of the grating. (**h**) Taper formed during recrystallization of a fibre with an initial core diameter of 20 μm, made by applying stress to the fibre during laser heating (Scale bar, 200 μm).

The tendency of the elements to segregate can be overcome, as shown above, but it can also be utilized to make gradient composition structures by design. By establishing a melt zone that penetrated the full width of the fibre, followed by slow defocusing of the laser to reduce power density, localized and highly asymmetric high-germanium concentration features could be formed in the core. Figure 5a,b shows XCT of a ∼100 μm spot written onto the side of a 6 at% Ge fibre by this method. The concentration increases to a maximum of 35 at% Ge (as determined by EDX and Raman shifts) at the highest point. This corresponds to a refractive index change of 0.13 (ref. 58), and a bandgap change from ∼1.1 to 0.96 eV (ref. 59) large enough to make devices based on the electronic properties.

Models of directional recrystallization predict that the composition of the material can be altered by changing the solidification velocity, and the fibres are an ideal system to test and exploit this effect for the fabrication of compositional gratings. Two different modulations were performed: (a) interrupting the CO_2_ laser beam momentarily, which caused a reduction in the volume of the melt and an acceleration of the solidification front (Supplementary Movie 4); and (b) explicitly varying the velocity of the phase front by altering the scan speed of the laser, including a rapid deceleration segment. Figure 5c shows an XCT image of a fibre with a scan performed at 50 μm s^−1^, using a 4 s/0.04 s on/off cycle (Fig. 5d). This resulted in germanium-rich regions due to acceleration of the phase boundary that were ∼40 μm wide, with a spacing of 200 μm. The bright stripes have a germanium concentration about 3 at% higher than that of the background, as determined by EDX. The velocity of the trailing edge immediately after closing the laser shutter was not resolvable at the video frame rate available, but a lower bound of ∼250 μm s^−1^ was determined.

To test deceleration effects, the stepper motor controlling the laser position was programmed to execute a saw-tooth velocity sweep between 0 and 200 μm s^−1^ with a repeat distance of 100 μm. Although the contrast is not as high, XCT clearly reveals the presence of Si-rich regions corresponding to the reset/deceleration from 200 μm s^−1^ to zero. Fig. 5e shows an intensity profile derived from the XCT image in Fig. 5f, while Fig. 5g shows the programmed velocity profile.

The heating of the glass to melt the core and the high temperatures attained allow structuring of the glass cladding as well as the core. In Fig. 5h, we illustrate one advantage of CO_2_ laser processing over a laser with a wavelength absorbed by the core; the softened fibres can be tapered to reduce the core diameter to a few μm. Similar tapers have been used to observe non-linear optical effects in silicon fibres^60^. We believe that cores down to 1 μm can be readily obtained, and possibly single-mode fibres can be obtained if the core remains continuous during the drawing process.

### Strain and orientation

Laser annealing of semiconductor core fibres using above-bandgap radiation, absorbed by the core rather than the silica, results in large strain effects^36^, making a comparison to the CO_2_ heating mechanism interesting. In addition, one of the most exciting prospects for germanium alloys is the possibility of efficient light emission under distortion of the lattice^61^,^62^,^63^. For the determination of strain, combining Raman and EDX data allows separation of the effects of strain and composition with a spatial resolution of ∼4 μm. While X-ray diffraction can be used for precise strain measurements via Bragg peak shifts, in SiGe microwires such analysis is complicated by the fact that the radial peak broadening is the convoluted effect of the SiGe composition gradients, the instrumental resolution, and the strain.

**Figure 6:**
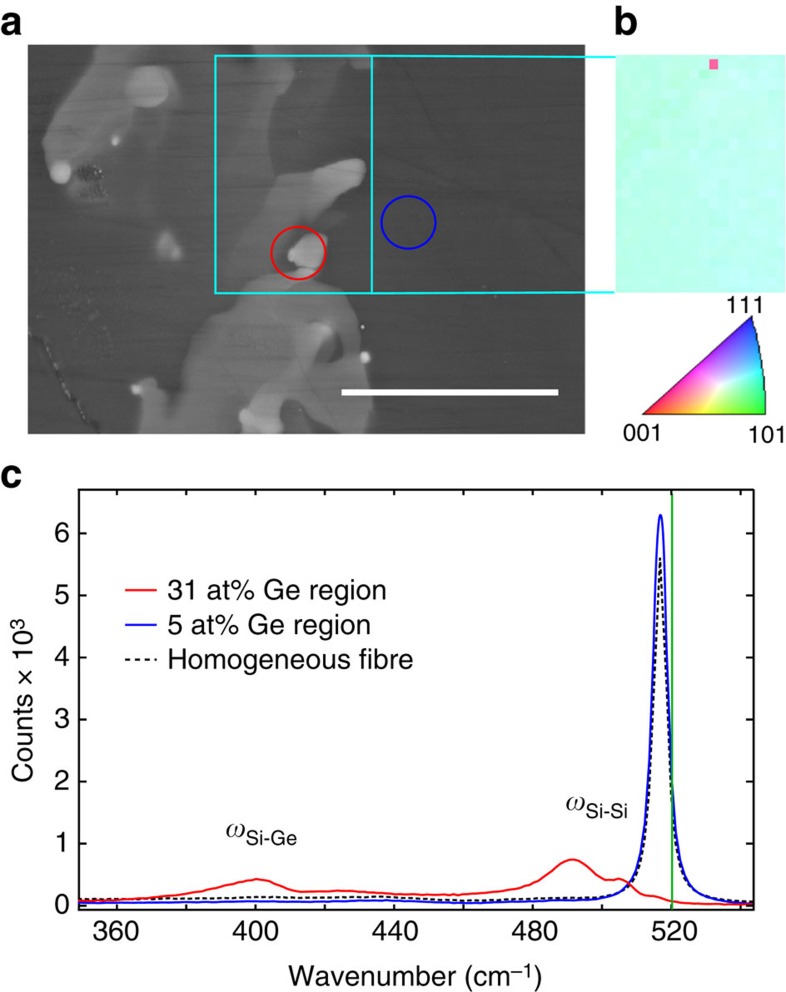
Compositional variations in untreated fibres. (**a**) BSE micrograph of a region with severe compositional inhomogeneity, with red and blue circles indicating Ge-rich and Ge-poor regions. Scale bar, 10 μm. (**b**) EBSD crystallographic map of the region inside the turquoise box, with the uniform colour indicating a single crystalline orientation despite large variations in composition. (**c**) Raman spectra of the regions in the circles; red (Ge-rich) and blue (Ge-poor). The dashed black curve is the result from a homogenized fibre, and the vertical green line is the shift measured for the Si–Si mode on a reference silicon wafer.

We examined the Raman spectra of the 6 at% alloy fibre samples to determine the magnitude and sign of any residual strain. There are three vibrational modes used for analysis of SiGe: the Si–Si mode at 500–520 cm^−1^, Si–Ge at around 400 cm^−1^ and Ge–Ge at approximately 290 cm^−1^; of these, the first two gave signals adequate for analysis. These modes are shifted by both composition and strain^64^,^65^, and if the germanium concentration, *x*, is measured by EDX^66^, the strain, *ɛ* can be determined from:


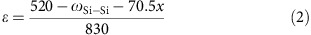


where *ω*_Si–Si_ is the measured Si–Si peak position in cm^−1^. Figure 6a shows a BSE image for an inhomogeneous region in one of the 6 at% Ge overall composition fibres, with two Raman analysis locations circled; these regions were chosen because they represent the extremes of composition observed in the as-drawn fibres. Figure 6b shows the crystal orientation map within the turqoise outline, as measured by EBSD, displaying a nearly constant crystal orientation despite large compositional variations.

EDX measurements from a germanium-poor region, marked by the blue ring, gave a composition of *x*_EDX_=0.054±0.005. With the measured Si–Si mode frequency, *ω*_Si–Si_=516.4 cm^−1^, equation (2) yields a tensile strain value of 0.037%, which is zero within the uncertainty of the measurement.

For the germanium-rich region marked by a red ring in Fig. 6a, the composition can be determined both by Raman deconvolution^66^ and EDX. Calculating the composition from Raman alone gives *x*≈0.31±0.01, in agreement with EDX measurements (*x*_EDX_≈0.34±0.03) from the same region. The values of ω_Si–Si_ and ω_Si–Ge_ were determined by peak fitting to be 490.6 and 400.2 cm^−1^, respectively for the germanium-rich region, indicating a tensile strain of 0.91%. This significant strain, associated with the high-germanium content regions, is attributed to differential shrinkage in the core after solidification, with the higher expansion coefficient of germanium-rich material resulting in larger strain. Additional Raman data, taken stepwise across a fibre, are shown in Supplementary Fig. 12.

Analysis performed for recrystallized fibres gave results similar to the germanium-poor region in the as-drawn fibre, with a minimal Si–Ge peak, and zero residual strain to within the accuracy of the measurements.

## Discussion

We have demonstrated that localized laser thermal annealing for heat treatment of SiGe alloy core fibres is a powerful technique for modifying the composition and crystal structure of the core. The fibre geometry allows for control and simultaneous observation of the crystallization process in addition to a range of post-processing analyses. The small heated volume, efficient convective cooling and the quasi one-dimensional geometry of the fibres made unusually large temperature gradients (∼10^4^ K cm^−1^) at the solidification interface possible. This, combined with the large diffusion coefficient associated with the high peak temperatures attained, could be used to promote uniform composition in the recrystallized core. Planar solidification fronts were observed at growth velocities that are 1–3 orders of magnitude larger than those reported for bulk samples, but still 100 times smaller than would be needed for in-line processing of fibre given the core diameters and temperature gradients studied so far. The critical velocity was observed to be greater for the fibres with smaller (10 μm) cores. This diameter is less than the facet lengths observed in two-dimensional recrystallization of SiGe^31^, and this suggests that the planar growth front may be stabilized by capillarity effects^48^. In both the large and small diameter fibres, increasing the germanium content in the fibres from 6 at% to 40 at% with a fixed temperature gradient resulted in a significant decrease in the critical growth velocity, consistent with the Tiller formulation. Fabrication of sub-micron-core fibres, where capillarity effects and larger thermal gradients are possible may permit the recrystallization of fibres in line during the drawing process by increasing the critical velocity. The limiting factor is likely to be the heat removal process required to maintain suitable temperature gradients. If sufficient cooling is possible, partitionless solidification^67^,^68^,^69^,^70^ may be observed, avoiding the compositional undercooling problems entirely.

In addition to the processing desired for applications requiring a homogeneous composition, the laser treatment and the segregation tendency can be used to create non-uniform concentration profiles. The method was used for the direct inscription of microscopic compositional features in optoelectronic fibres. Fine spatial control over the growth velocity and melt zone geometry can be used to create rewritable periodic structures in the fibre, suggesting that waveguide dispersion modification and Bragg grating mirrors are possible.

As an example, silicon is a well-known material for terahertz waveguiding, and fibres with cores sizes of 130 μm and a grating period of 45 μm could be used for filtres and splitters in this important spectral region. Small concentrations of germanium are adequate to make strong Bragg gratings (for example, the 3% germanium variation observed here would lead to Δ*n*∼0.018).

Modification of the dispersion characteristics can also occur far from the Bragg wavelength, suggesting potential non-linear infrared applications such as mode coupling and supercontinuum generation^71^,^72^,^73^,^74^. Compared with planar waveguides, the fibre geometry would permit easier coupling of these sources to optical measurement systems.

It should be noted that the 40 μm grating features demonstrated here are significantly smaller than both the laser beam waist (160 μm) and the width of the melt zone (500 μm). This is because the deposited composition is primarily a function of the local velocity at the solidification front and the diffusion properties in the interface region. Further studies are needed to determine the shortest period grating that can be written.

Interestingly, scanned CO_2_ laser systems have been introduced recently for localized diffusion treatments of silicon circuits, and our results suggest that this equipment could be employed for controlled crystallization of encapsulated amorphous SiGe films. This would permit direct integration of high-mobility circuit elements^30^ with minimal germanium concentration gradients and reduced heating of the wafer, in a two-dimensional equivalent of our process.

This work introduces SiGe-core fibres and demonstrates the potential for laser heat treatment of semiconductor alloy optical fibres, both for fundamental materials studies and for direct formation of microscale compositional features. Unstable growth at high solidification velocity (for example, during the drawing process), an increase in the critical velocity with reduced dimensions, and suppression of nucleation at velocities approaching the critical value have all been observed. The technique employed here is generally applicable to additional materials and geometries.

## Methods

### Fibre fabrication

The majority of the experiments were performed on fibres with a core composition of Si_0.94_Ge_0.06_ and a silica glass cladding made in a single-fibre-drawing operation. Fibres were fabricated by placing silicon and germanium pieces into a thick-walled silica tube (preform) closed at one end, and heating the assembly to ∼1,950 °C. The semiconductor alloy melted, and when the glass softened and was drawn downwards, a coaxial fibre was formed with the alloy in the centre. Rapid diffusion within the small volume of the semiconductor liquid assured complete mixing. In the drawing process, many metres of fibre were produced as the diameter of the preform was reduced from ∼30 to 1 mm and the core reduced from 4 mm to ∼130 μm diameter; no change in average composition was detectable from one end of the draw to the other. The drawing speed was ∼1.3 m min^−1^. Fibres with smaller core diameters were made by loading the resulting 1 mm composite cane into a silica capillary and redrawing to a smaller diameter using oxygen–hydrogen torch heating. Samples with 25 at% and 40 at% Ge were made using 4–6 mm diameter preforms, flame heating to ∼2,000 °C and drawing at 1–2  m s^−1^. In some cases the silicon and germanium were pre-melted under vacuum for these smaller samples, as the time in the molten phase is short. All fibres were made with a CaOH interface modifier, as described in Nordstrand *et al*.^47^

### Laser treatment

Laser recrystallization was performed using an radio frequency modulated 28 W CO_2_ laser (*λ*=10.6 μm) that was focused to a spot size of 166 μm (Gaussian FWHM) with a 63.5 mm focal length ZnSe lens. Power adjustments were made by changing the radio frequency modulation duty cycle, and the heat zone width was controlled by defocusing the spot on the fibre by 5–10 mm. The laser was scanned at the desired velocity using stepper motor controllers. A CCD camera (Thorlabs DCU224C) was placed perpendicular to the laser beam for imaging the melt zone. Narrowband filters centred at 514 and 632 nm were placed in front of the camera for the measurements used in temperature estimation (Supplementary Note 1; Supplementary Figs 3 and 4).

### Electron microanalysis

The composition of the fibre cores was investigated by SEM on polished and planarized cross-sections. BSE and energy dispersive X-ray spectrometry (EDX) measurements were performed using a Hitachi TM3000 SEM for the large core SiGe fibres, and a Hitachi S-5500 S(T)EM for the small core fibres. Before imaging, the samples were cleaned in acetone and isopropanol sonication baths for 5 min, and a 4 nm Pt/Pd film was deposited using a Cressington 208 HR B sputter coater to reduce charging effects from the glass.

EBSD analysis was performed on a ZEISS Ultra with a beam current of 60 μA and an acceleration voltage of 20 kV to minimize charging, since the samples could not be coated.

### Optical characterization

The optical transmission losses of the fibres were measured using the standard cut-back technique. The fibre was mounted in a larger host capillary and polished using routine fibre preparation methods. To ensure that two-photon absorption was avoided, low power continuous wave laser diodes were used at an output power of 2 mW. The measurements were undertaken at two wavelengths, with a 1,550 nm (Tunics-plus) system and with a 2,000 nm (Thorlabs, FPL2000S) source. Each laser was launched into the core of the fibre using a 0.65 NA anti-reflection coated fused silica objective that was selected to ensure the light was coupled predominantly into the fundamental core mode. The output of the fibre was imaged using an Electrophysics 7290 IR camera to confirm that transmission occurred only through the semiconductor core, thus providing a good indication of the bulk SiGe material quality. The coupling was optimized using a set of Thorlabs Nanomax stages. The measurements were performed on sections of fibre that were ∼1 cm in length. A 1 mm section of fibre was removed for each cut-back measurement and this was repeated four times for each fibre to ensure the integrity of the measurement.

### X-ray analysis and imaging

X-ray diffraction measurements were performed using the general-angle X-ray scattering set-up at the Norwegian Resource Centre for X-ray Scattering and Imaging at NTNU (Supplementary Note 3; Supplementary Figs 8–11). A molybdenum microfocus source with a source current of 1 mA and acceleration voltage of 50 kV was used. The X-ray radiation was monochromatized (Mo *K*_α_, *λ*=0.71 Å) and focused to about 0.2 mm diameter using multilayer focusing optics from Xenocs SA. A Huber cross slit was used to clean the beam. The diffraction patterns were acquired with an acquisition time of 60 s using a Pilatus 1 M area detector from Dectris AG having 981 × 1,043 pixels with a pixel size of 172 μm × 172 μm. The detector was positioned 134 mm downstream of the sample, which yields a *q* range of 0.002–4.0 Å^−1^, where the magnitude of the scattering vector is defined by *q=*4*π*sin*θ*/*λ*, 2*θ* being the angle between the incident and scattered beams. The microwire samples were mounted with their long axis parallel to the vertical rotation axis and either translated axially, or rotated by an angle *φ* about their long axis. Integrated patterns were obtained by summing patterns collected in 1° angular increments over a *φ* range of 180° (Supplementary Figs 1,9). The software SimDiffraction^75^ was used for data treatment.

X-ray tomography was performed using an XT H 225 ST micro-CT system from Nikon Metrology NV, using a tungsten target, a source current of 47 μA and acceleration voltage of 145 kV. The high-resolution images were acquired using a Perkin Elmer 1620 flat panel detector with a pixel size of 200 μm × 200 μm and 2,048 × 2,048 pixels. The microwire samples were mounted with their long axis parallel to the vertical tomography axis and scanned in the range 0° to 360°.

### Data availability

All data are available from the corresponding author on request.

## Additional information

**How to cite this article:** Coucheron, D. A. *et al*. Laser recrystallization and inscription of compositional microstructures in crystalline SiGe-core fibres. *Nat. Commun.* **7,** 13265 doi: 10.1038/ncomms13265 (2016).

## Supplementary Material

Supplementary Information Supplementary Figures 1-12, Supplementary Notes 1-3 and Supplementary References.

Supplementary Movie 1 25 at% Ge as-drawn fibre showing dendritic structures. The video is made from XCT by creating surfaces of constant grey value corresponding to constant compositions. The grey values are then scanned to show the structure as the silicon concentration increases. In this highly undercooled fibre, a silicon rich primary dendrite has grown rapidly in the axial direction, then developed lateral arms that forced Ge towards the outside of the fibre. This video reveals the three-dimensional structure of the dendrites.

Supplementary Movie 2 6 at% Ge as-drawn fibre showing coring structures. The video is made from XCT by extracting surfaces of constant grey value corresponding to Ge content, and scanning through the values (from low to high Ge concentration) to show the evolution of the shape with increasing Ge content. Initially the glass coating is "stripped away", then the high-Si regions are removed to reveal the residual Ge-rich cores. The apparent "texture" after the glass is stripped is an indication of the non-uniformity of the surface composition; a similar structure is observed when fibres are chemically etched in HF, due to differential etch rates for the Si- and Ge- rich regions.

Supplementary Movie 3 Partial melting of the core. Translation of a 6 at% Ge fibre through the CO2 induced heat zone during partial melting of the core: Ge-rich regions in the untreated zone (to the right) melt at lower temperature, and flow towards the high temperature region, forming a melt-zone that is Ge rich. The left hand side has already been exposed to the temperature gradient and no Ge-rich material remains. As a result, no material is seen flowing from left to right in the frame.

Supplementary Movie 4 Modulated laser power. Changes to the melt zone, during translation along a fibre, caused by a series of 0.04s interruptions in the CO2 laser beam. The melt zone decreases in volume, and the trailing (solidification) boundary accelerates dramatically. This leads to an increase in the concentration of Ge in both the melt zone and the material deposited during the acceleration. The velocity increase is proportionately larger than the volume decrease, and thus dominates the effect of this treatment.

## Figures and Tables

**Figure 1:**
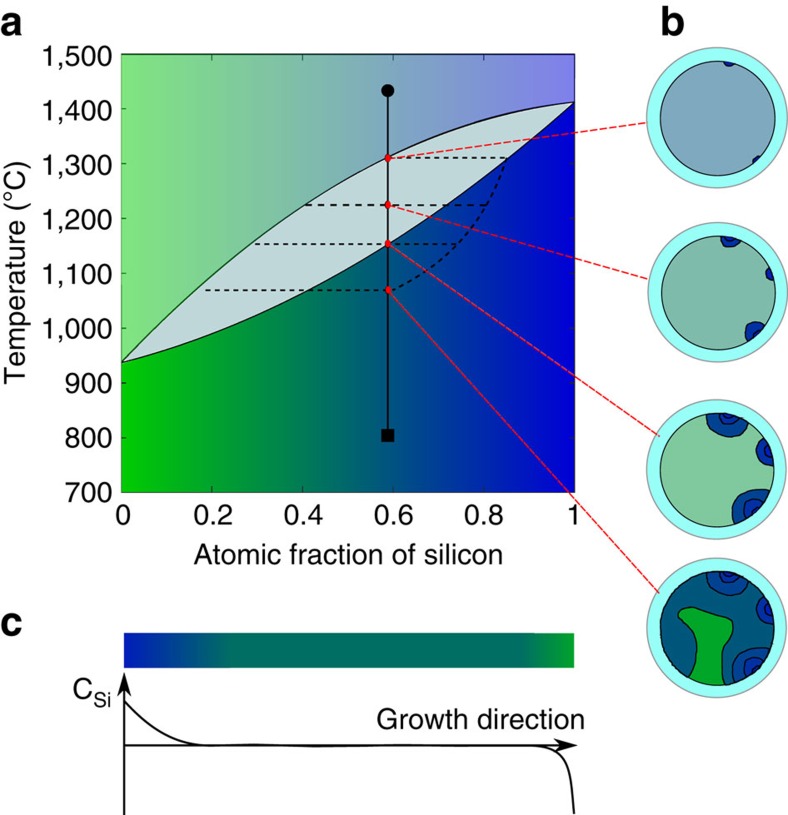
Compositional segregation of SiGe. (**a**) The phase diagram of SiGe, with (**b**) a schematic illustration of non-equilibrium cooling in fibre cross-sections resulting in residual Ge-rich regions (green). (**c**) Compositional variation along the growth axis during directional recrystallization of a thin rod.
