# Supplementary material for: Laser recrystallization and inscription of compositional microstructures in crystalline SiGe-core fibres
Source: Nat Commun. 2016 Oct 24;7:13265. doi: 10.1038/ncomms13265 (PMC5079062; doi:10.1038/ncomms13265)
Supplement: Supplementary Information — Supplementary Figures 1-12, Supplementary Notes 1-3 and Supplementary References. [file ncomms13265-s1.pdf]

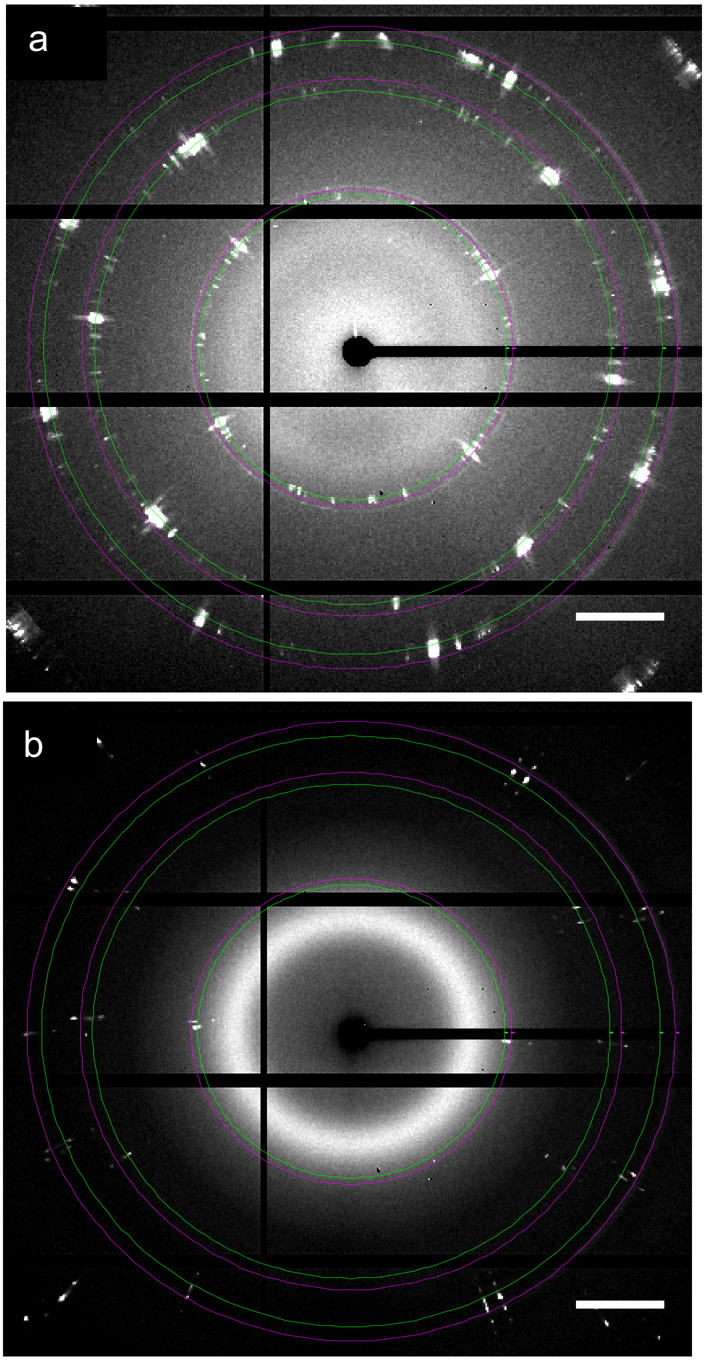

Supplementary Figure 1

Integrated X-ray diffraction patterns over 120° sample rotation. Magenta circles indicate the tabulated powder diffraction rings {111}, {220} and {311} for silicon, green for germanium. (a) 25 at% Ge, (b) 6 at% Ge. Both samples exhibit a high degree of order and orientation. The high-Ge sample is polycrystalline, with a preferential orientation of the <001> axes parallel to the fibre. In contrast, the low-Ge sample has only two crystals in the particular scattering volume shown here, and these differ in orientation by only a few degrees. Interestingly, the latter diffraction pattern has an entirely different symmetry, consistent with <110> oriented along the axis of the fibre. Scale bars 1 Å^{-1}.

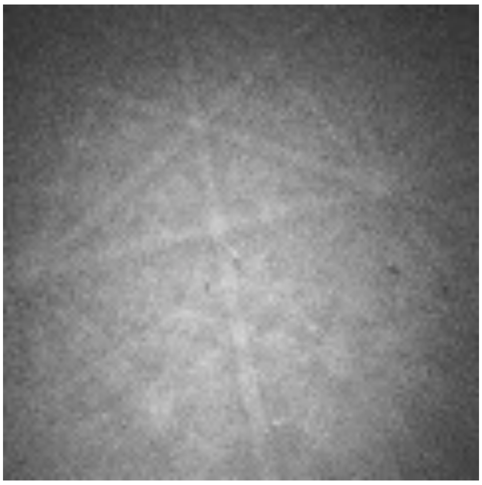

Supplementary Figure 2
As-drawn fibre crystallinity. Representative electron backscattered diffraction pattern from an as-drawn 6 at% Ge fibre; the entire fibre cross section showed the same pattern (see Fig. 6 in the main text for a map of the orientation). Brightness and contrast were each increased by 40% over the original image.

.

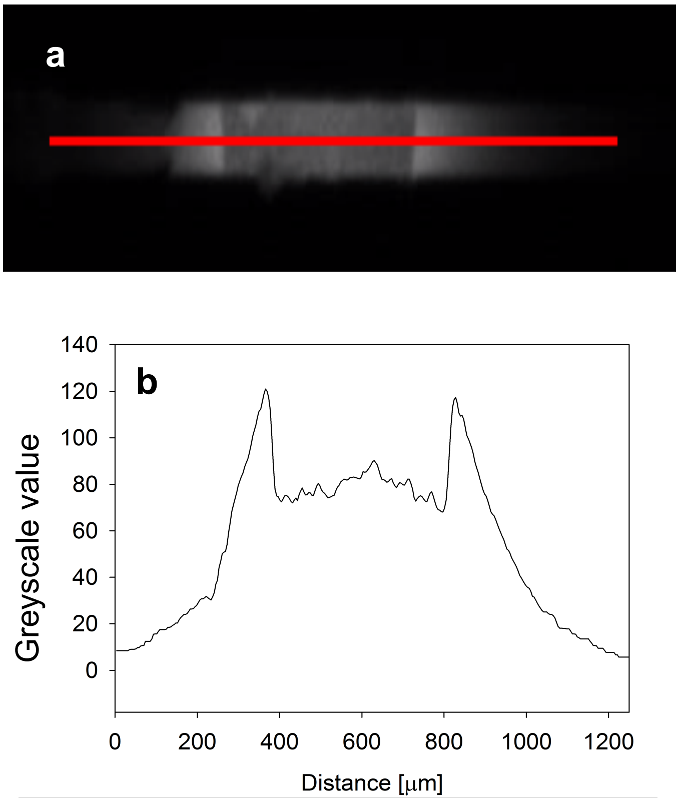

Supplementary Figure 3

Emission profile at 514nm. Image of fibre melt zone using a 514 nm narrow band filter (a) and a greyscale value plot from the red line (b). A sharp decrease in greyscale value in seen at the solid-liquid interface due to a difference in emissivity of the two phases. Noise in the central region is due to emission from particles in the interface layer.

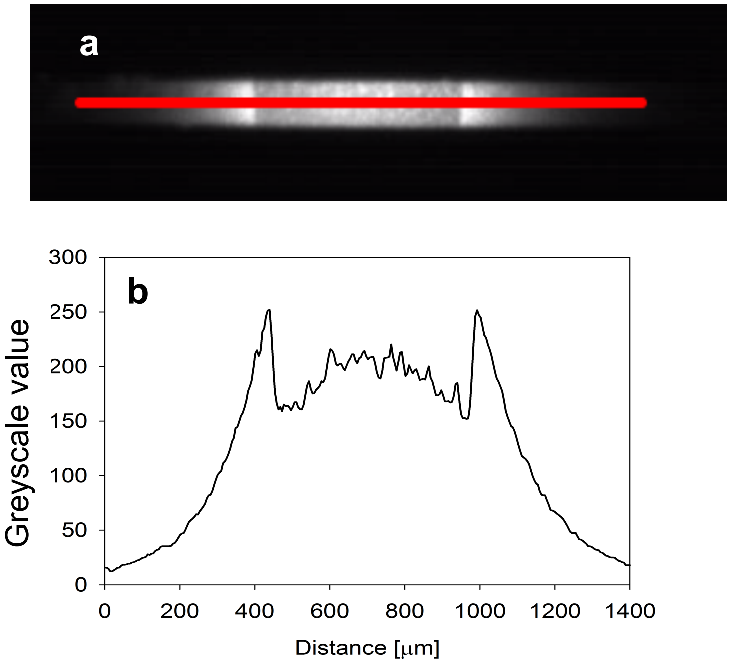

Supplementary Figure 4

Emission at 633nm Image of the fibre melt zone (a) through a 633 nm narrow band filter and (b)corresponding greyscale values from the red line

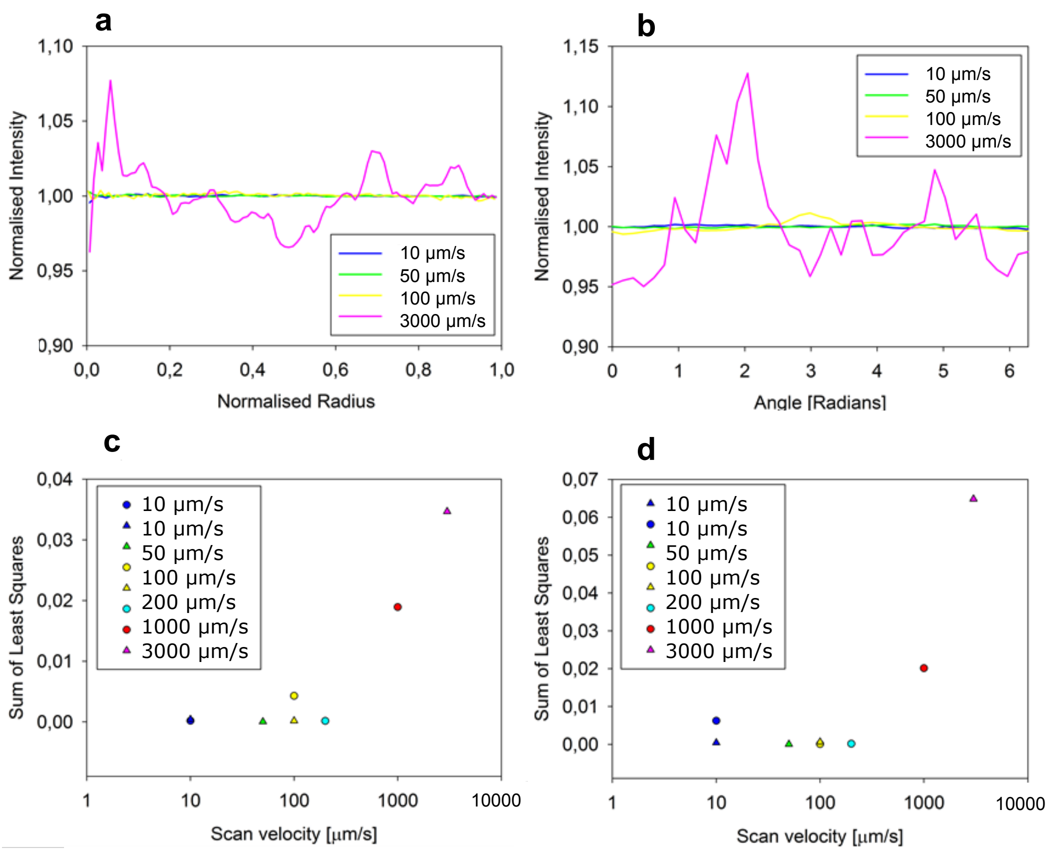

Supplementary Figure 5

Compositional uniformity tests (a) Integrated intensity over concentric rings as a function of normalized fibre radius, (b) Integrated intensity over angular sections as a function of rotation around a fibre and resulting homogeneity as a function of laser scan speed for (c) radial case and (d) angular case, showing onset of inhomogeneity as the critical velocity of ~200-500 µm s^{-1} is exceeded.

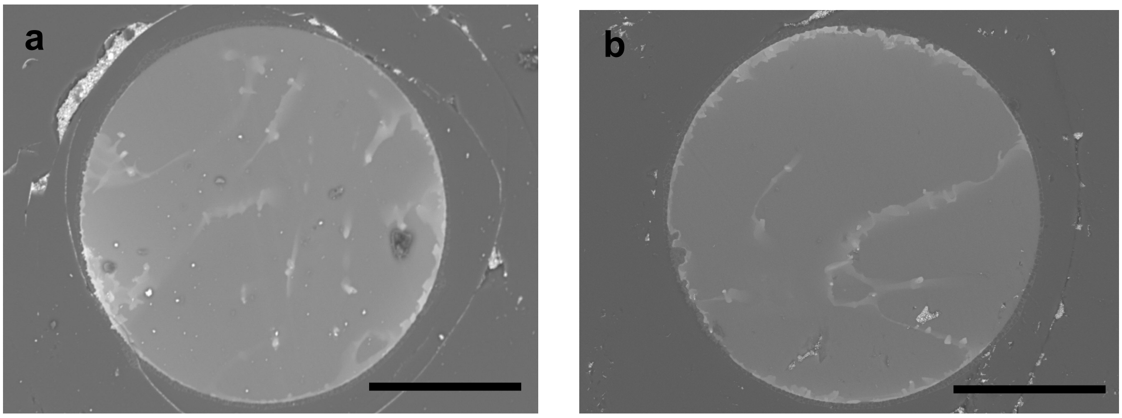

Supplementary Figure 6

Inhomogeneous compositions BSE SEM micrographs illustrating the inhomogeneous composition observed in a fiber scanned at 1000 µm s^{-1} (a) and an untreated fiber (b). Scale bars are 50 µm.

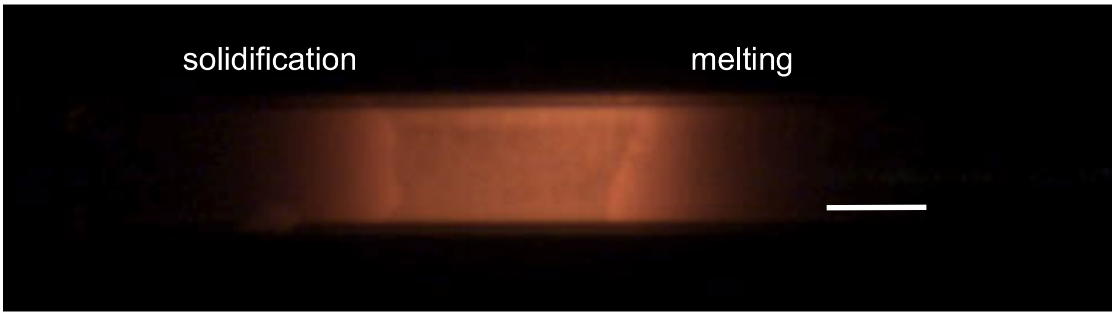

Supplementary Figure 7
High Ge content core Image extracted from video taken during annealing of a 250 µm core fibre with 40 at% Ge, translated at 10 µm s^{-1}, near the critical velocity. The left hand interface, where solidification occurs, exhibited instability, with significantly varying features and curvature, while the melting interface on the right maintained a constant shape. Scale bar is 200µm.

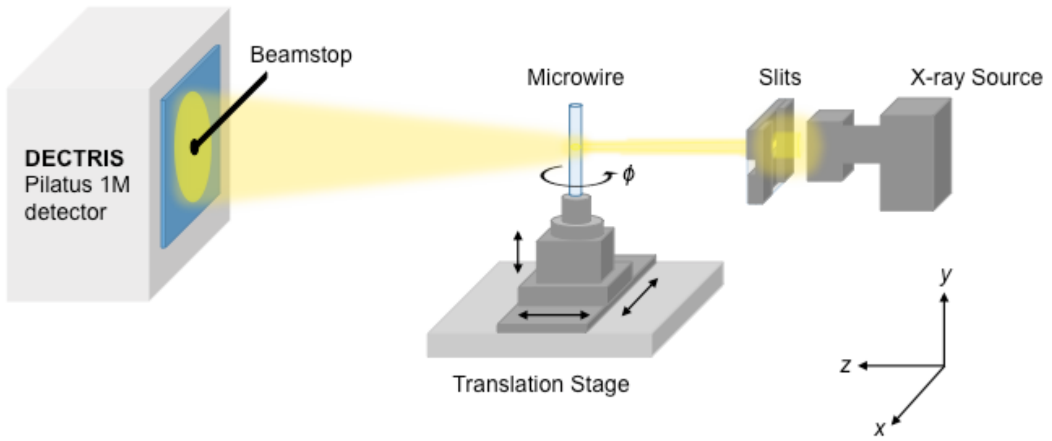

Supplementary Figure 8
X-Ray diffraction Sketch of the XRD setup.

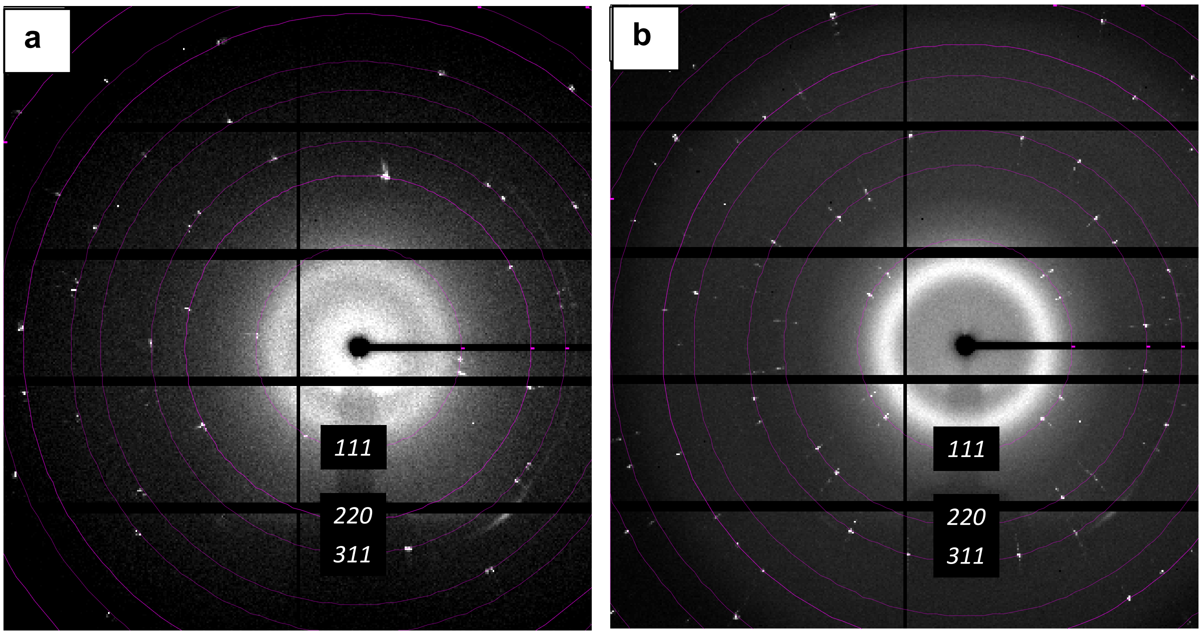

Supplementary Figure 9
Diffraction patterns integrated over a range of projection angles ϕ a) Pure Ge microwire, overlaid with the calculated powder diffraction rings for Ge. The Bragg peaks are sharp, with the radial width dominated by the instrumental resolution. b) Recrystallized SiGe (6 at% Ge) microwire, overlaid with the calculated powder diffraction rings for Si. In this case, the Bragg reflections in (b) are radially broadened, as expected for an inhomogeneous Si-Ge blend. The strong isotropic scattering at low q can be ascribed to the glass surrounding the semiconductor core.

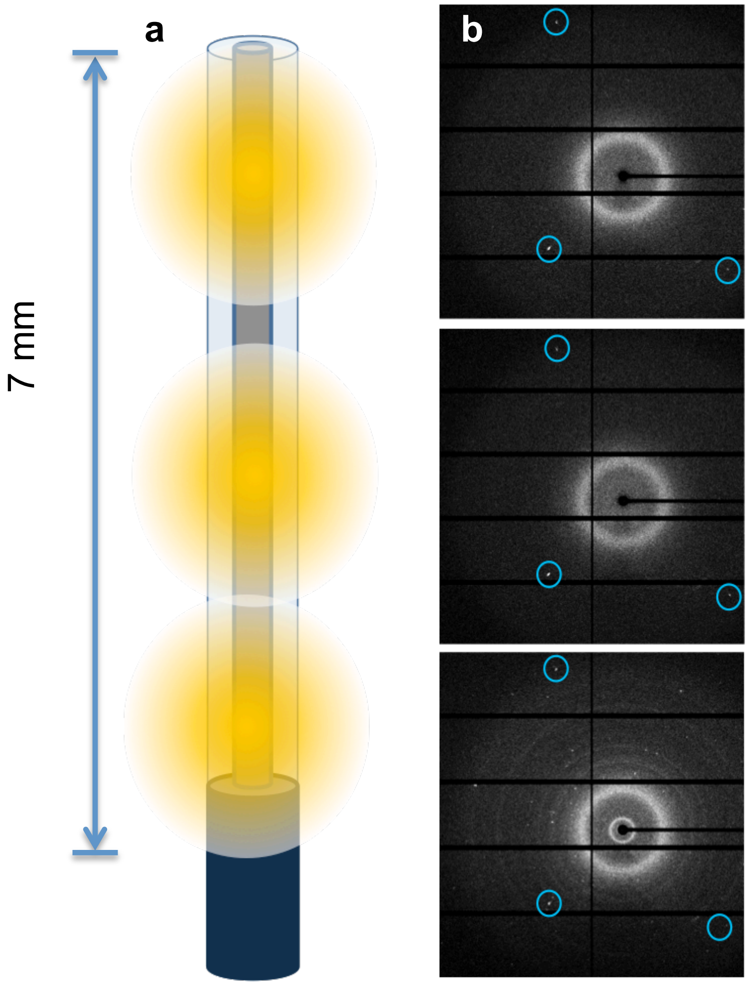

Supplementary Figure 10

Axial XRD scans (a) Schematic drawing of the recrystallized SiGe microwire. The yellow patches indicate the approximate X-ray beam position with spot size ≈ 200 µm. (b) The corresponding diffraction patterns acquired at the different axial positions, all obtained for the same sample orientation angle ϕ. The blue circles highlight the Bragg peaks. By slightly changing ϕ, these diffraction peaks disappeared and others appeared, as expected. Additional powder diffraction rings observed in the lowermost diffraction pattern are due to the plasticene used for mounting the microwire.

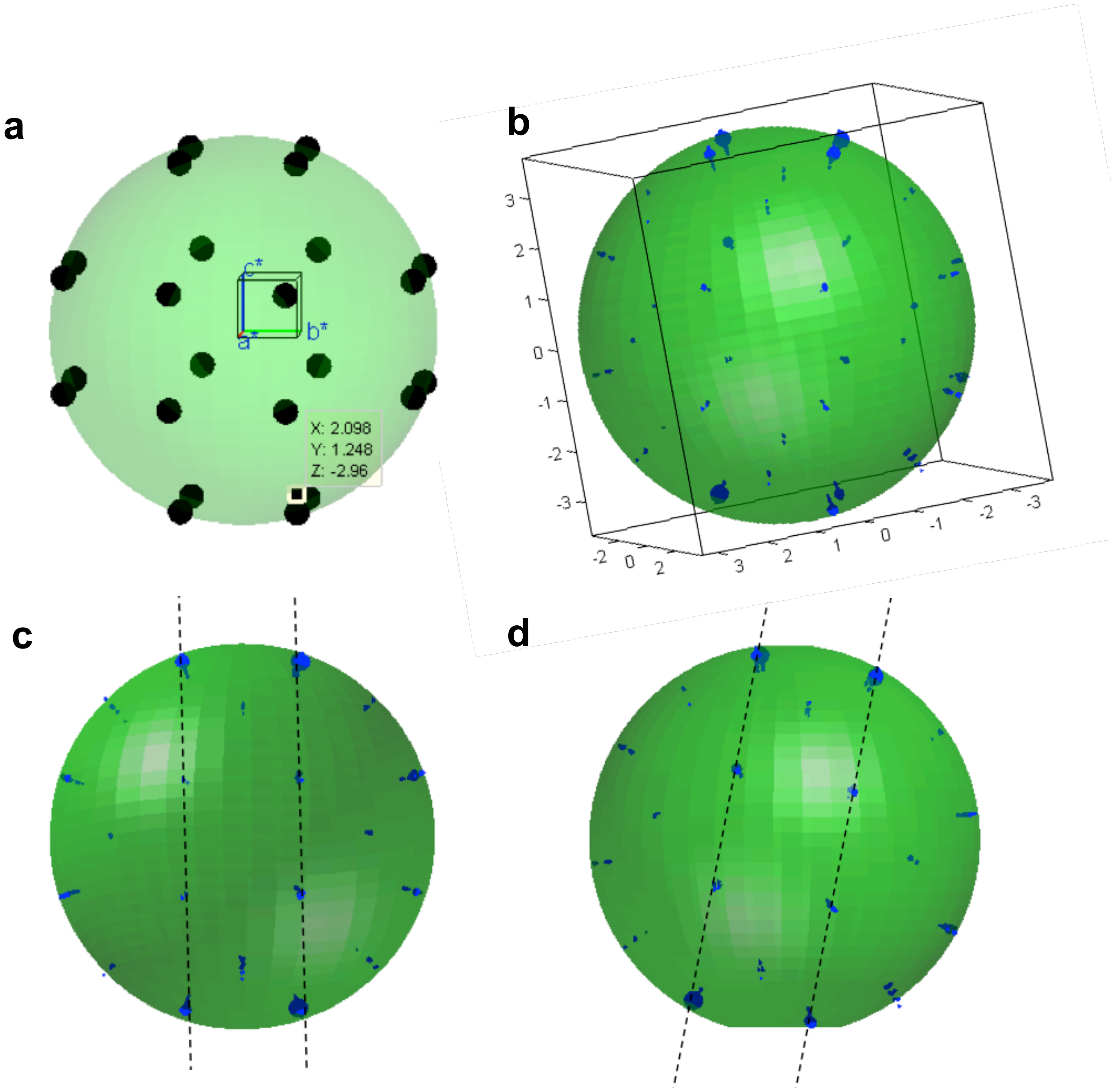

Supplementary Figure 11
3-d reconstruction Reciprocal space structure for the {311} family of reflections. The green spheres indicate a surface of constant $q = 3.84$ Å^{-1}. (a) The ideal reciprocal space structure for Si, oriented with the unit cell *c*-axis vertical. (b) Reconstructed reciprocal space for the {311} family of reflections based on the experimental data for the single-crystalline sample, suitably oriented for comparisons with (a). Note the striking resemblance with the theoretical pattern, confirming that the sample is single-crystalline and highly oriented. (The additional weaker Bragg peaks at highly symmetric positions are high-*q* tails of the {220} reflections.) (c),(d) Views of 3D reciprocal space along the [100] and [010] zone axes, respectively. The unit cell is tilted by about 12° about the *b*-axis.

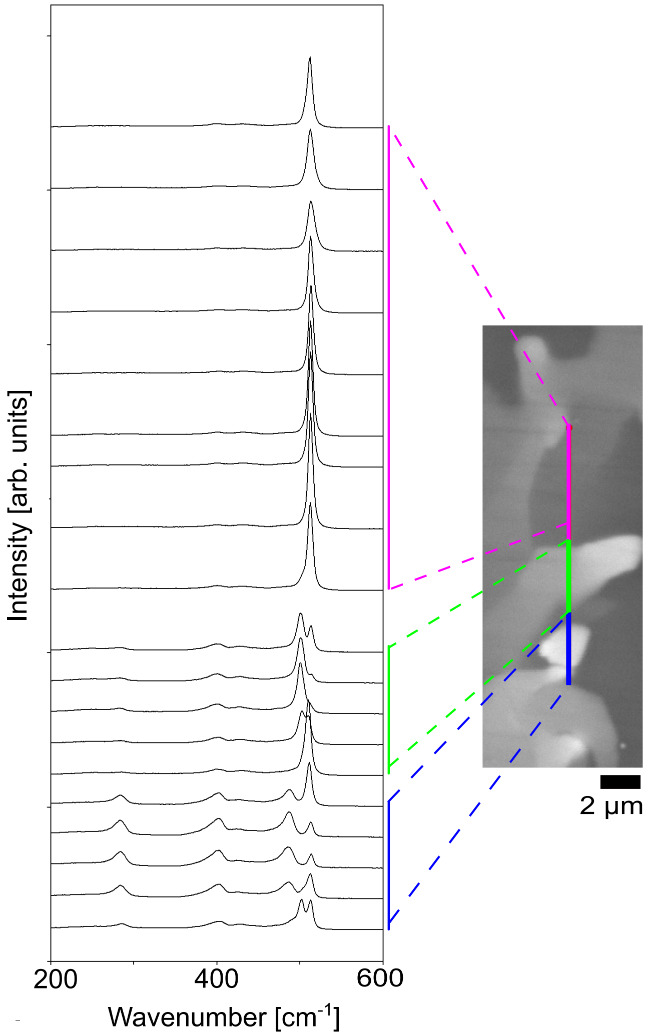

Supplementary Figure 12
Raman line scan Raman spectra taken at 1 µm intervals along the colored line shown at right overlaid on a BSE image of an untreated fibre. The vertical offset of each spectrum is arbitrarily chosen to make the main features visible. The blue section has Ge concentration of ~30%, the green section has an intermediate composition, and the pink section has a Ge concentration of 5%. In transition regions, where contributions from two different compositions are sampled, a dual peak is seen for silicon near 520 cm^{-1}. Strain values (indicated by shifts in the silicon peak position) are constant within each composition region.

Supplementary Note 1
Estimation of Temperature Gradient

The temperature gradient in the liquid SiGe adjacent to the solidification front, which is key to determining the likelihood of undercooling and hence the critical velocity (see Supplementary Note 2), was estimated for CO_2-laser recrystallization experiments using the measured emission intensity profiles and the blackbody distribution. A 514 or 633 nm narrow band filter was inserted in front of the B&W camera during annealing with standard illumination, at a scan velocity of 10 μm s^{-1} Stills from the monochromatic videos (Supplementary Fig. 3 and 4), were exported to ImageJ[1] to make plots of the relative intensity vs. position. While there are no suitable literature values for the emissivity (ε) of SiGe alloys, both silicon and germanium have near-constant values[2] of ε in the solid phase (~0.6, 0.53 respectively[3]) and in the liquid phase (0.23[2], 0.17[4]) over the temperature range of interest. The large discontinuity in the profile at the interface between the molten zone and the solid SiGe is due to this difference in emissivity at the solid-liquid interface. The ratio of the emissivity values observed in the images, based on the peak and trough at the interface, was ~1.7, comparable to the ratios for both pure silicon and pure Ge. The melting temperature provided a single calibration point, and was estimated for the fibre core using the approximate composition of the liquid in conjunction with the equilibrium phase diagram.

The Ge content of the melt is higher than the fibre average due to migration of Ge-rich material to the melt zone (as shown in Supplementary Video 3) and due to the preferential segregation of silicon into the solid phase, as indicated by the phase diagram. A concentration of approximately 9 at% Ge was estimated using the width over which Ge was gathered, giving a melting temperature of 1673K. By considering the ratio between the highest intensity value in the melt and the value at the interface

(~1.3), assuming a constant emissivity in the melt and linear response in the detector, the temperature difference can be estimated using the Planck distribution:

$$B_\lambda(\lambda, T) = \frac{2hc^2\varepsilon}{\lambda^5}\left(e^{\frac{hc}{\lambda k_\mathrm{B} T}} - 1\right)^{-1} \quad (1)$$

where ε is the emissivity, h the Planck constant, c the speed of light, λ the wavelength, T the temperature and k_B the Boltzmann constant. Taking the intensity value in Supplementary Fig. 3 at the melting point *B(514 nm, 1673* K) and solving for T at an intensity 1.3 times as great gives a maximum temperature of 1983 K. Dividing by the distance from the interface to the maximum yields an upper bound of 1.4×10^4 K cm^{-1} for the temperature gradient .

The same procedure was performed using a 633 nm narrow band filter. A greyscale value ratio of ~1.24 is observed for the frame presented in Supplementary Fig. 4. Solving for T_{max} gives a thermal gradient of 1.5×10^4 K cm^{-1}, in reasonable agreement given the noise levels in the images.

Supplementary Note 2

Critical Velocity

The breakdown of a planar solid-liquid growth interface during unidirectional crystallization was first described by Chalmers and Rutter in 1953[5]. Tiller *et al.*[6] further advanced the theory by formulating a condition for a stable planar interface, henceforth referred to as the Tiller criterion or critical velocity (*cf.* main text).

For solidification velocities above a critical value, a layer of constitutionally undercooled liquid causes a breakdown of the planar growth interface and results in inhomogeneous composition in dilute binary alloys.

The resulting compositional inhomogeneity, indicative of exceeding the critical velocity, was investigated by cross-sectional BSE imaging of the fibres, as discussed

below. For 130 μm core 6 at% Ge fibres, recrystallization speeds up to 200 μm s^{-1} resulted in homogeneous composition. With higher concentrations (40 at% Ge), fibres with a core diameter of 250 ±40 μm demonstrated a dramatic reduction in critical growth velocity to 10 μm s^{-1} in the presence of similar temperature gradients (in agreement with the Tiller model). However, for the 6 at% Ge fibres with a diameter of ~15 μm, a homogeneous distribution was observed at the highest tested growth rates of 1000 μm s^{-1}. Similar small-diameter fibres with a composition of 40 ± 2.5 at% Ge had a critical growth velocity, of ~100 μm s^{-1}, ten times higher than for the thick fibres, indicating the stabilizing role of small core dimension at both concentrations.

An estimate of the theoretical critical growth velocity for the 6 at% Ge fibres can be made using the thermal gradient determined in Supplementary Note 1. The liquidus and solidus lines of the SiGe phase diagram are parameterized as:

$$T_L(\mathrm{K}) = 1685 - 80x - 395x^2 \quad (2)$$

$$T_S(\mathrm{K}) = 1685 - 738x + 263x^2 \quad (3)$$

The slope of the liquidus can then be determined by differentiating equation (2). Solving equation (2) and (3) with respect to x for T=1673 K (the interface temperature) and inserting into

$$k = \frac{x_S}{x_L} \quad (4)$$

yields the segregation coefficient. The diffusion constant[7] is taken to be constant at the interface and equal to 28 × 10^{-5} cm^2 s^{-1}, Finally, inserting all values into (1) gives a critical velocity of v_c=164.6 μm s^{-1}.

The problems in realising homogeneous growth of SiGe are typically ascribed to constitutional undercooling due to the large miscibility gap (see Fig. 1a, main text). The severity of the constitutional undercooling depends on the composition of the melt, but also the growth velocity of the phase front, as a higher growth velocity will suppress solute diffusion into the liquid. The Tiller criterion for inhomogeneous growth during unidirectional solidification is still being used today to predict critical growth rates. However, Mullins and Sekerka[8] presented a model that also considers the effect of the difference in thermal conductivity between the phases, the temperature gradients in the phases, the latent heat released, the curvature effects on equilibrium concentration at the interface and capillarity (solid-liquid interface energy) and thus the lateral dimensions of the interface.

In their model, capillarity and high temperature gradients stabilize the phase front. Thus, higher growth velocities can be used while still suppressing inhomogeneous growth in small dimensions and with large temperature gradients. Additionally, Yim and Dismukes[9] have pointed out that strong thermal gradients will enhance thermal diffusion and further stabilize the phase front.

Experiments were performed with fibre cores of ~130 μm and ~15 μm to see whether a difference in the critical velocity could be measured as a function of radius. The compositional uniformity across the polished fibre cross-sections indicated whether the critical velocity had been exceeded. The Ge content in electron micrographs of polished cross-sections gives greyscale contrast in backscattered electron (BSE) imaging, and automated analysis of these images was performed using a MATLAB® script. Images were taken with identical microscope settings and were not processed prior to analysis. Polishing minimized topological contrast in the BSE signal, leaving only atomic number contrast. Edge detection was performed to

determine the core/cladding interface, the radius, *R*, and the position of the core centre. Greyscale values were integrated in evenly spaced annuli of width *R*/100 and were normalized by the average greyscale value for the image to provide a quantitative metric of the fibre inhomogeneity, as seen in Supplementary Fig. 5a. A similar procedure with angular slices of size $2\pi/40$ was performed to visualize the angular distribution, as seen in Supplementary Fig.5 b. The sum of least squares (SLS) for the radial and angular distributions gives a single-value indication of the homogeneity of a fiber, with a compositionally uniform fiber having a SLS of 0. Plotting the resulting SLS for fibres recrystallized at different velocities, the critical value for the onset of inhomogeneous growth can be determined. Supplementary Fig. 5c presents the radial SLS, and Supplementary Fig. 5d shows the angular SLS for all tested growth velocities of the 6 at% Ge fibers with core diameters of 120 μm. There is a very distinct step between 200 and 1000 μm s^{-1}.

Manual investigation of SEM images of an untreated sample and a sample treated at 1000 μm s^{-1} reveals a very similar compositional distribution, as seen in Supplementary Fig. 6. This suggests that either the critical growth rate was reached and inhomogeneous growth occurs, or possibly, insufficient power was available for melting the cores at these high rates.

Supplementary Note 3
X-ray diffraction

3.1 Experimental details

The text description of the experimental details is presented in the main manuscript and Supplementary Fig. 8 shows the geometry used.

3.2 X-ray diffraction analysis

The microwires were measured still encased in glass. X-ray diffraction patterns were collected i) as function of axial position along the microwire length for a chosen angle ϕ, and, ii) as a function of angle ϕ for rotations about the microwire long axis at selected axial positions.

Phase identification

The diffraction patterns acquired at selected axial positions with 1° steps in ϕ were summed to obtain rotationally integrated diffraction patterns, thus being similar to the classical so-called "rotating crystal method". The summed diffraction patterns were compared with the calculated powder diffraction rings of the well-known diamond cubic unit cells for Ge and Si. Examples of integrated diffraction patterns are shown in Supplementary Fig. 9 for a pure Ge microwire and for a recrystallized (100 μm s^{-1} scan rate) SiGe (6 at% Ge) microwire.

The diffraction patterns for the pure Ge microwire were, as expected, in agreement with the diamond cubic unit cell of Ge with a = 5.66 Å. The diffraction data for recrystallized SiGe exhibited radial broadening centred near the diamond cubic unit cell of Si having a = 5.43 Å.

Axial scans

With a beam spot size of 200 μm the spatial resolution was sufficient to observe single or polycrystalline regions along the length of the microwire. XRD measurements on the recrystallized SiGe microwire (100 μm s^{-1} scan rate) at different axial positions, obtained for the same sample orientation angle ϕ, are shown in Supplementary Fig. 10. Diffraction patterns obtained millimetres apart exhibit the same diffraction peaks, revealing longitudinal uniformity of the crystal structure and hence crystallographic coherence over several millimetres. Other samples did not exhibit this coherency, signifying that those samples were polycrystalline with

crystalline domains smaller than the volume probed by the X-ray beam.

Rotation scans and 3D reciprocal space analysis

Having obtained diffraction data for a wide range of projection angles ϕ, three-dimensional reconstructions of reciprocal space were calculated. The symmetries of different Bragg reflections were studied, as shown in Supplementary Fig. 11 for the {311} family of reflections, which has q = 3.84 $Å^{-1}$ and a multiplicity of 24. Supplementary Fig. 11a and 11b show the ideal reciprocal space structure and the acquired experimental data at approximately the same sample orientation. Supplementary Fig. 11c shows the experimental {311} reflections viewed along the [100] zone axis having the reflections parallel to the fiber axis. Notably, as shown in Supplementary Fig. 11d, when viewed along the [010] zone axis the Bragg reflections are tilted at an angle of 12±2° with respect to the microwire principal axis. This ~12° tilt angle matches the tilt of the elongated solidified structures seen in the CT images (cf. main text). Assuming that the cross section can be considered smooth and uniform across the fiber diameter, the observed tilt would correspond to a vicinal (501) surface, giving a crystallographic cut angle of arctan(1/5) = 11.3°. We note that EBSD data (Supplementary Fig. 2) indicate that physically cut cross-sections exhibit an orientation between the (101) and (111) planes. Referring also to Supplementary Fig. 1, it is clear that different fibres exhibit different preferred crystal orientations. While the nucleating crystallite is likely to be randomly oriented, the XCT images also indicate that in some cases (cf. Fig. 3a in the main text), the fibre geometry guides and reorients the subsequent crystal growth.

Supplementary References

1. Schneider, C. A., Rasband, W. S. & Eliceiri, K. W. NIH Image to ImageJ: 25 years of image analysis. *Nat. Methods* **9,** 671–675 (2012).
2. Kawamura, H., Fukuyama, H., Watanabe, M. & Hibiya, T. Normal spectral emissivity of undercooled liquid silicon. *Meas. Sci. Technol.* **16,** 386–393 (2005).
3. Allen, F. G. Emissivity at 0.65 Micron of Silicon and Germanium at High Temperatures. *J. Appl. Phys.* **28,** 1510–1511 (1957).
4. Rhim, W.-K. & Ishikawa, T. Thermophysical Properties of Molten Germanium Measured by a High-Temperature Electrostatic Levitator1. *Int. J. Thermophys.* **21,** 429–443 (2000).
5. Rutter, J. W. & Chalmers, B. A Prismatic Substructure Formed During Solidification of Metals. Can. J. Phys. 31, 15–39 (1953).
6. Tiller, W. ., Jackson, K. ., Rutter, J. . & Chalmers, B. The redistribution of solute atoms during the solidification of metals. Acta Metall. 1, 428–437 (1953).
7. Schilz, J. & Romanenko, V. N. Bulk growth of silicon-germanium solid solutions. *J. Mater. Sci. Mater. Electron.* **6,** 265–279 (1995).
8. Mullins, W. & Sekerka, R. Stability of Planar Interface During Solidification of Dilute Binary Alloy. J. Appl. Phys. 35, 444–& (1964).
9. Dismukes, J. P., Ekstrom, L. & Paff, R. J. Lattice Parameter and Density in Germanium-Silicon Alloys1. J. Phys. Chem. 68, 3021–3027 (1964).
